# Host natural killer immunity is a key indicator of permissiveness for donor cell engraftment in patients with severe combined immunodeficiency^☆^

**DOI:** 10.1016/j.jaci.2014.02.042

**Published:** 2014-06

**Authors:** Amel Hassan, Pamela Lee, Paraskevi Maggina, Jin Hua Xu, Diana Moreira, Mary Slatter, Zohreh Nademi, Austen Worth, Stuart Adams, Alison Jones, Catherine Cale, Zoe Allwood, Kanchan Rao, Robert Chiesa, Persis Amrolia, Hubert Gaspar, E. Graham Davies, Paul Veys, Andrew Gennery, Waseem Qasim

**Affiliations:** a Immunology and Bone Marrow Transplant Units, Great Ormond Street Hospital for Children, London, United Kingdom; b Cellular & Molecular Immunology, Institute of Child Health, University College London, London, United Kingdom; c Bone Marrow Transplant Unit, Great North Children's Hospital, Royal Victoria Infirmary, Newcastle upon Tyne, United Kingdom; d Institute of Cellular Medicine, University of Newcastle, Newcastle upon Tyne, United Kingdom

**Keywords:** Severe combined immunodeficiency, conditioning, natural killer cells, chimerism, engraftment, adenosine deaminase deficiency, ADA, Adenosine deaminase deficiency, allo-SCT, Allogeneic hematopoietic stem cell transplantation, JAK3, Janus kinase 3, MFD, Matched family donor, MSD, Matched sibling donor, NK, Natural killer, SCID, Severe combined immunodeficiency, TREC, T-cell receptor excision circle

## Abstract

**Background:**

Severe combined immunodeficiency (SCID) can be cured by using allogeneic hematopoietic stem cell transplantation, and the absence of host immunity often obviates the need for preconditioning. Depending on the underlying genetic defect and when blocks in differentiation occur during lymphocyte ontogeny, infants with SCID have absent or greatly reduced numbers of functional T cells. Natural killer (NK) cell populations are usually absent in the SCID-X1 and Janus kinase 3 forms of SCID and greatly reduced in adenosine deaminase deficiency SCID but often present in other forms of the disorder.

**Objective:**

To determine if SCID phenotypes indicate host permissiveness to donor cell engraftment.

**Methods:**

A retrospective data analysis considered whether host NK cells influenced donor T-cell engraftment, immune reconstitution, and long-term outcomes in children who had undergone nonconditioned allogeneic stem cell transplantation between 1990 and 2011 in the United Kingdom. Detailed analysis of T- and B-cell immune reconstitution and donor chimerism was compared between the NK^+^ (n = 24) and NK^−^ (n = 53) forms of SCID.

**Results:**

Overall, 77 children underwent transplantation, with survival of 90% in matched sibling donor/matched family donor transplants compared with 60% when alternative donors were used. Infants with NK^−^SCID were more likely to survive than NK^+^ recipients (87% vs 62%, *P* < .01) and had high-level donor T-cell chimerism with superior long-term recovery of CD4 T-cell immunity. Notably, 33% of children with NK^+^SCID required additional transplantation procedures compared with only 8% of children with NK^−^SCID (*P* < .005).

**Conclusions:**

NK^−^SCID disorders are highly permissive for donor T-cell engraftment without preconditioning, whereas the presence of NK cells is a strong indicator that preparative conditioning is required for engraftment of T-cell precursors capable of supporting robust T-cell reconstitution.

Severe combined immunodeficiencies (SCIDs) are a heterogeneous group of genetic disorders with common clinical phenotypes presenting in early infancy with serious or recurrent infections and failure to thrive. For more than 40 years, allogeneic hematopoietic stem cell transplantation (allo-SCT) has provided curative therapy for these disorders.^1^ There has been longstanding debate and controversy around how and when to best perform transplantations in infants given a diagnosis of SCID.^2^ Conventionally, conditioning comprising myeloablative or submyeloablative chemotherapy is used in patients undergoing allo-SCT to both eradicate host cellular immunity and empty the bone marrow niche in readiness for donor stem cell engraftment. In small infants conditioning might carry notable morbidity and mortality, and thus it has been long argued that in the absence of host cellular immunity, sufficient donor cell engraftment can be achieved without ablative conditioning. Some centers strongly advocate infusion of unmanipulated donor grafts, arguing that in the HLA matched family donor (MFD) setting, sufficient T-cell engraftment can be achieved without preconditioning and that this can sustain long-term immune recovery.^3-5^ In general, although these patients might not have significant levels of multilineage stem cell engraftment, adequate donor T-cell engraftment in combination with successful seeding of T-cell precursor niches sufficient to maintain thymopoiesis can support long-lived immune recovery. Engraftment of B-cell or myeloid precursors is usually low or absent, and although a number of children will recover antibody production, many will require immunoglobulin replacement therapy for life.^2^ In the absence of an HLA identical donor, stringently T cell–depleted mismatched hematopoietic stem cell grafts can also mediate sustained thymopoiesis, although in the absence of mature donor T cells, recovery is slow and takes many months.

Analyses of SCID cohorts undergoing transplantation in Europe and North America^4,6-9^ have given rise to the concept of permissive and nonpermissive host environments, and the presence of host natural killer (NK) immunity might be an important determinant in considering which infants require preconditioning. Aside from donor matching, there are 2 additional and interrelated aspects of host NK immunity that might be critical in determining successful outcomes in these infants. First, innate cellular immunity mediated by macrophages and NK cells might be a barrier against donor cell engraftment.^10^ Data from animal studies suggest that NK cell immunity plays a crucial role in the rejection of allogeneic cells, particularly in the HLA-mismatched setting.^11^ Second, underlying genetic defects largely determine when lymphoid differentiation arrests during ontogeny, and this in turn directly influences whether niches for T-cell and NK cell precursors are occupied. Thus SCID disorders caused by defective common gamma chain (SCID-X1)^12^ and Janus kinase 3 (JAK3) SCID^13^ have circulating B cells but no T-cell or NK cell immunity (T^−^B^+^NK^−^), reflecting a defect that impedes common T/NK precursor development.

In other SCID disorders in which T-cell developmental arrest occurs at a later stage of differentiation, NK development is unaffected, and presumably the common T/NK precursor niche is occupied, which might result in competition during engraftment. SCID caused by adenosine deaminase (ADA) deficiency usually affects multiple lineages and is associated with NK deficiency (although less profound), and NK cell numbers might recover if children are detoxified with enzyme replacement therapy with PEG-ADA ahead of allo-SCT.

Previous surveys of transplantation outcomes for SCID considered the importance of the SCID phenotype based on the presence or absence of B cells, and T^−^B^+^ hosts fared better than T^−^B^−^ recipients.^14^ The importance of host NK cell immunity was widely suspected, but incomplete recipient characterization precluded detailed analysis. Here we report the United Kingdom experience of nonconditioned allo-SCT for SCID disorders and, for the first time, confirm that the presence of host NK immunity is a key indicator of whether the host SCID environment is likely to be permissive for donor cell engraftment.

## Methods

### Patients' characteristics

Between 1990 and 2011, 77 infants (52 male) with SCID underwent allo-SCT without preconditioning at 2 United Kingdom pediatric centers commissioned to undertake such procedures (see Table E1 in this article's Online Repository at www.jacionline.org). All were T-cell deficient, with absent T cells or reduced T-cell numbers with absent or severely impaired proliferation responses to the mitogen PHA.

Age at transplantation was similar for the groups and ranged from 1 week to 17 months, with a median age of 3 months and a median follow-up of 3070 days for the NK^+^ group and 3001 days for the NK^−^ group (range, 760-7300 days). All children received antimicrobial prophylaxis and immunoglobulin replacement therapy from the time of diagnosis. SCID phenotypes were determined on the basis of flow cytometry for T cells (CD3), B cells (CD19), and NK cells (CD16/CD56) and, where indicated, had defined molecular defects on the basis of abnormal or absent protein expression, metabolite analysis, or genetic mutations. Patients were grouped for analysis based on the presence (>100 × 10^6^/L) or absence (<100 × 10^6^/L) of NK cells at the time of transplantation. This threshold reflects the lower limit of normal for age range of NK cell numbers in healthy infants. Bone marrow or cord blood grafts were infused (n = 49) in the matched sibling donor (MSD) or fully HLA MFD setting. Bone marrow or cord blood was also infused from 11 HLA-matched unrelated donors. Haploidentical grafts were enriched for stem cells by using CliniMacs CD34 selection (n = 17). No prophylaxis against graft-versus-host disease was given to 35 infants, and the remainder received cyclosporine alone or in combination with mycophenolate mofetil, prednisolone, or both.

Event-free survival was defined as survival without resorting to second procedures, and where indicated, second procedures were undertaken with conditioning. Details are provided in Table E1, where second procedures are highlighted in gray below data for the primary infusion.

### Engraftment and chimerism

Whole blood, granulocyte, or mononuclear cells were subjected to chimerism analysis in all subjects. Where indicated, lineage-specific chimerism was also determined for CD3 (T cells), CD15 (myeloid lineage), and CD19 (B cells) after magnetic bead selection from peripheral blood by using an AutoMACS Pro-Separator (Miltenyi Biotec, Bergisch Gladbach, Germany). The PowerPlex 16 system (Promega, Southampton, United Kingdom) was used to PCR amplify 16 short tandem repeat loci in these patient samples. The PCR products were then analyzed by using an AB3130 Genetic Analyser with Gene Mapper v4.0 software (Life Technologies, Carlsbad, Calif).

### Statistical methods

Kaplan-Meier curves were used to analyze survival figures. The log-rank (Mantel-Cox) and Gehan-Breslow-Wilcoxon tests were used to compare survival between different groups. Logistic regression was performed with SPSS software (SPSS, Chicago, Ill) to identify determinants of survival after hematopoietic stem cell transplantation in different groups.

## Results

### Survival of infants with SCID after allo-SCT

**Fig 1:**
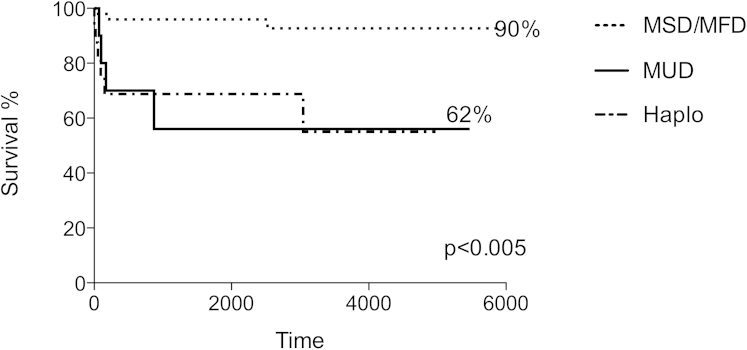
On the basis of donor type, overall survival of infants with SCID after nonconditioned allo-SCT with MSD or MFD donors (49/77 infusions) was 90%, and that after matched unrelated grafts *(MUD)* and haploidentical *(Haplo)* transplantations (28/77 infusions) was 62%.

Overall survival after nonconditioned allo-SCT procedures was 81%, with 90% survival after MSD or MFD infusions (49/77 procedures, Fig 1). Matched unrelated grafts and haploidentical transplantations (28/77 procedures) had less favorable outcomes, with survival of 62% in both groups. For comparison, over the same period, overall survival after conditioned allo-SCT for SCID was 72% (n = 148), although confounding differences in donor type and graft sources prevented controlled analysis against the nonconditioned cohort. Most notably, 112 of 148 conditioned procedures involved unrelated donor or mismatched donor grafts, whereas the majority of nonconditioned procedures used matched sibling or family donors.

**Fig E1:**
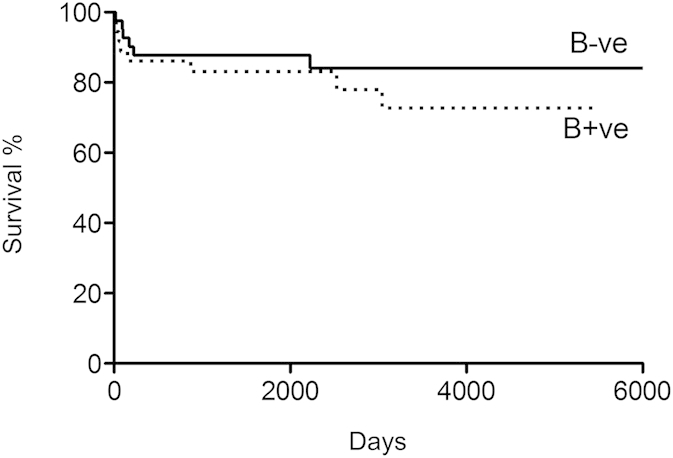


**Fig 2:**
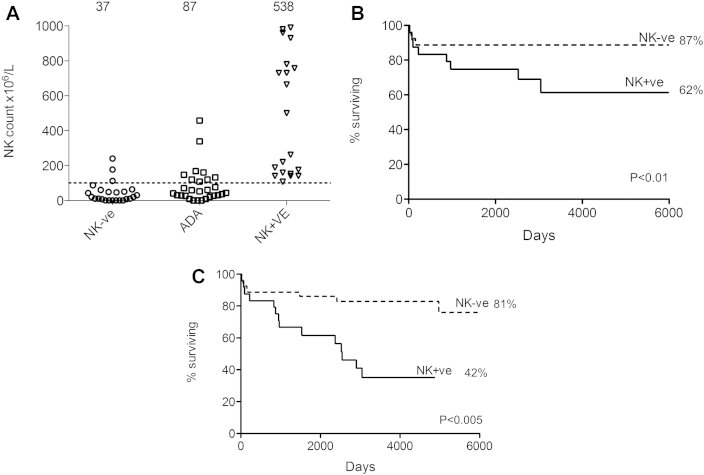
**A,** Primary NK deficiency was defined as less than 100 × 10^6^/L, and NK cell counts were low in children with T^−^B^+^ SCID (SCID-X1 and Jak3 deficiency) and ADA deficiency. SCID disorders with normal NK cell numbers (mean, 538 × 10^6^/L) were heterogeneous and included both T^−^B^+^ SCID and T^−^B^−^ SCID disorders. **B,** On the basis of the absence or presence of NK cells, overall survival after nonconditioned transplantations for NK^−^SCID was 87% compared with 62% for NK^+^SCID (*P* < .01). **C,** Event-free survival, which was defined as survival without the need for a subsequent procedure, was 81% for patients with NK^−^SCID compared with 42% in the NK^+^ group (*P* < .005). *NK+ve*, NK^+^; *NK-ve*, NK^−^.

In contrast to previously described analyses based on the presence or absence of host B cells,^14^ we found that transplantation survival for both patients with T^−^B^+^ and those with T^−^B^−^ SCID were similar (see Fig E1 in this article's Online Repository at www.jacionline.org). We next considered whether the presence or absence of NK cells could provide an indication of which SCID disorders are amenable to nonconditioned transplantation. Primary NK cell deficiency was defined as less than 100 × 10^6^/L, which is less than the normal range for infants aged 0 to 6 months (Fig 2, *A*). This group of infants were all T^−^B^+^, with underlying mutations of the common γ-chain or JAK3, giving rise to arrested T-cell and NK cell development. In our cohort NK numbers in patients with ADA-SCID were reduced at presentation (mean, 87 × 10^6^/L), and thus data for patients with ADA-SCID were pooled with the NK^−^SCID group for overall analyses (n = 53), although reconstitution data were also considered independently given that a number of these patients received enzyme replacement therapy ahead of transplantation, which might have supported detoxification and partial lymphocyte recovery. Patients with NK^+^SCID disorders (n = 24) had intact NK numbers at diagnosis (mean, 550 × 10^6^/L) and were genetically heterogeneous, with defined (RAG1, n = 7; Artemis, n = 2; IL-7 receptor, n = 4; and RMRP, n = 1) and undefined underlying disorders (n = 10).

Survival after nonconditioned transplantations for NK^−^SCID was 87% compared with 62% for NK^+^SCID (*P* < .01; Fig 2, *B*). Further analysis of the NK^+^ group found that the 9 infants with known defects of VDJ recombination (RAG1, RAG2, and Artemis) had poorer survival (56%) compared with those with the other 15 NK^+^ disorders in whom survival was 71%. Importantly, the overall number of children who required additional procedures was 33% in the NK^+^SCID group compared with only 8% of children with NK^−^SCID. This resulted in an event-free survival of 81% for patients with NK^−^SCID compared with 42% for patients with NK^+^SCID (*P* < .005; Fig 2, *C*). For these second transplantations, conditioning was administered in 6 children. In terms of complications, there was no difference in the incidence of graft-versus-host disease (grade II or greater) or viral reactivations between the NK^+^ and NK^−^ groups (Table E1).

### T-cell reconstitution and donor chimerism after nonconditioned allo-SCT

**Fig 3:**
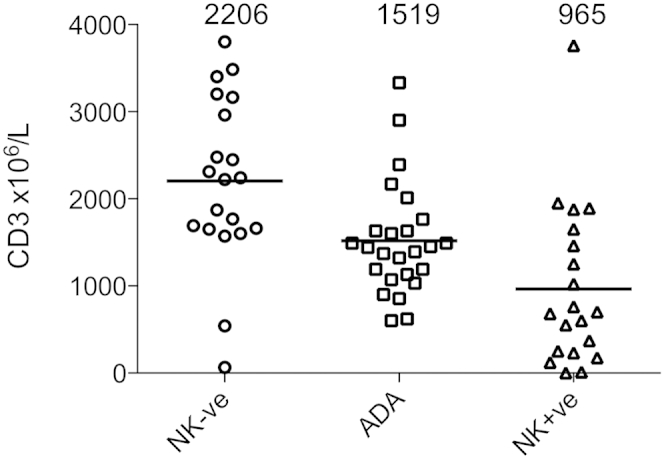
CD3 T-cell recovery was superior for patients with NK^−^SCID, with normalization of counts in most children, in contrast to those with NK^+^SCID disorders. *NK+ve*, NK^+^; *NK-ve*, NK^−^.

**Fig 4:**
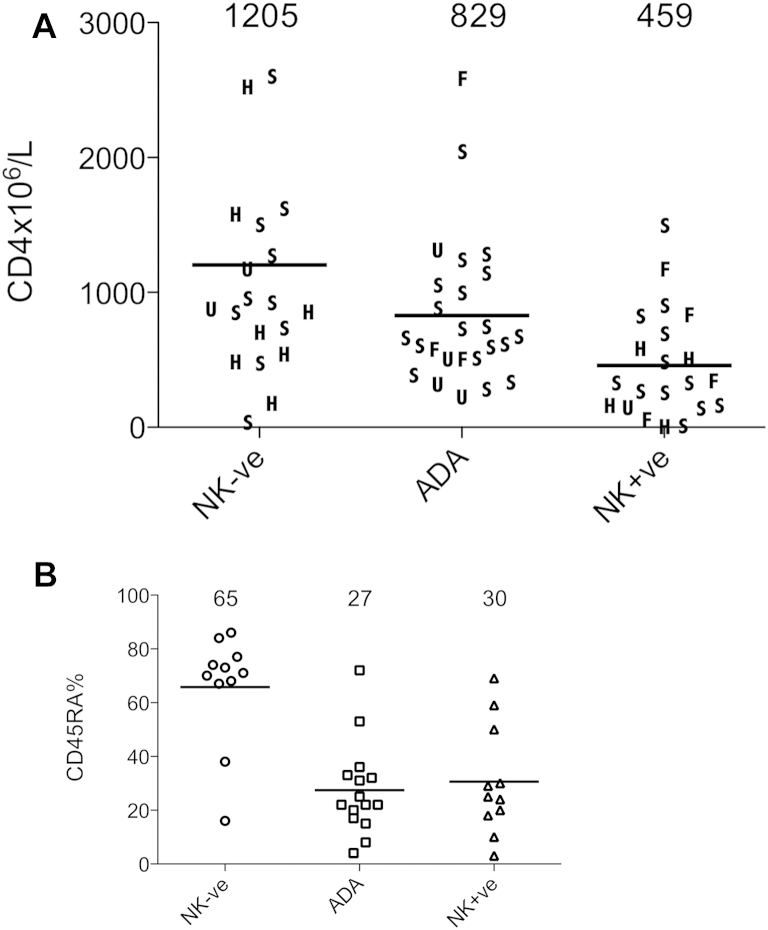
**A,** CD4 T-cell count recovery for NK^+^SCID was poorer than for NK^−^SCID or ADA-SCID. The source of the donor graft is highlighted. *F*, MFD; *H*, haploidentical; *S*, MSD; *U*, matched unrelated donor. **B,** Naive CD4 T-cell recovery provided an indication of thymopoiesis, and recovery was greatest in patients with NK^−^SCID, intermediate in patients with ADA-SCID, and low in patients with NK^+^SCID. *NK+ve*, NK^+^; *NK-ve*, NK^−^.

At the most recent follow-up (2-20 years after transplantation), the majority of children with NK^−^SCID disorders had peripheral blood CD3 T-cell counts within normal ranges for age. In contrast, T-cell recovery after nonconditioned allo-SCT for NK^+^SCID variants was suboptimal for 13 of 19 evaluable children. Interestingly, subgroup analysis found that recovery in patients with ADA-SCID was poorer than for those with NK^−^SCID, although superior than that for patients with NK^+^SCID (Fig 3). Analysis of CD4 T-cell counts uncovered a similar pattern of cellular recovery for the 3 groups, with immune recovery for different graft sources (MSD, MFD, matched unrelated donor, and haploidentical), as highlighted in Fig 4, *A*.

**Fig E2:**
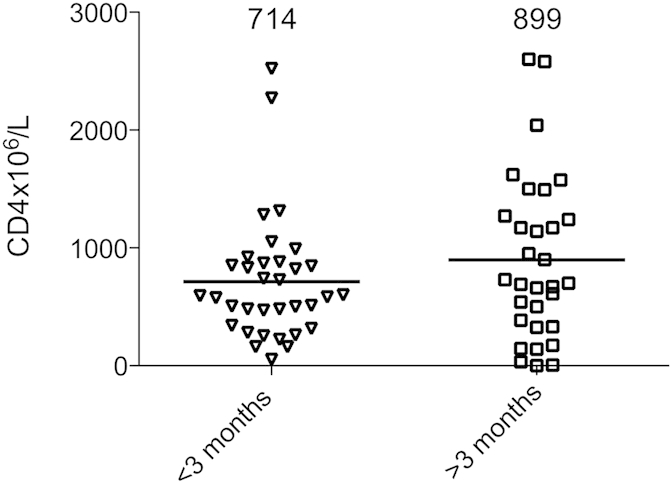


Where data for naive CD4 T-cell recovery were available, we found that 12 of 19 children with NK^−^SCID compared with 3 of 16 children with NK^+^SCID had normal proportions of naive CD4 T cells for age (*P* < .005). Within the NK^−^ group, children with ADA-SCID were again found to have intermediate levels of naive T-cell recovery (Fig 4, *B*). Additional evidence of thymic output based on quantification of T-cell receptor excision circles (TRECs) was available on 30 children and was consistent with superior thymopoiesis in the NK^−^SCID group, with a median of 37,521 TRECs per million T cells compared with 1,705 TRECs/million T cells in the NK^+^SCID group. There was no difference in CD4 T-cell recovery for infants treated before or after 3 months of age (see Fig E2 in this article's Online Repository at www.jacionline.org).

### Lineage-specific donor chimerism after nonconditioned transplantation

**Fig E3:**
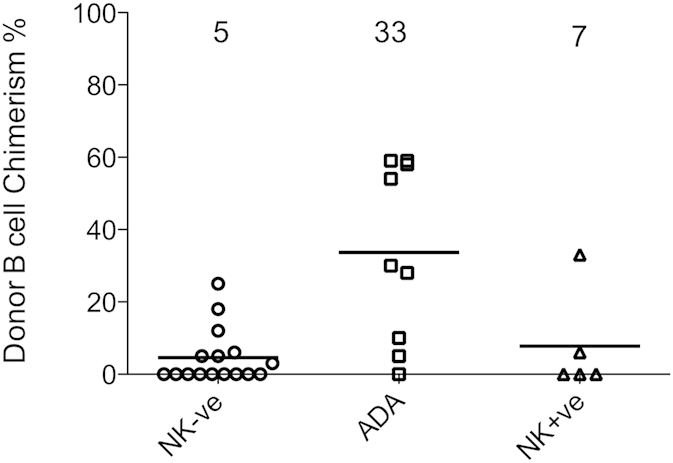


**Fig E4:**
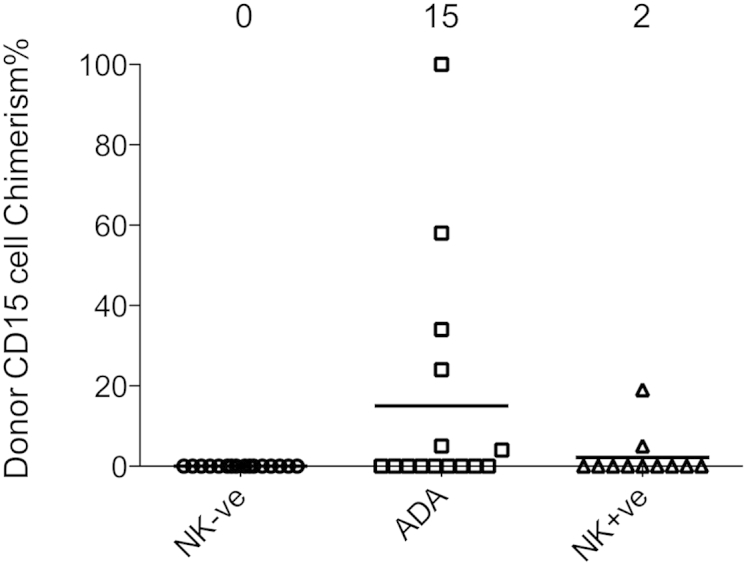


**Fig 5:**
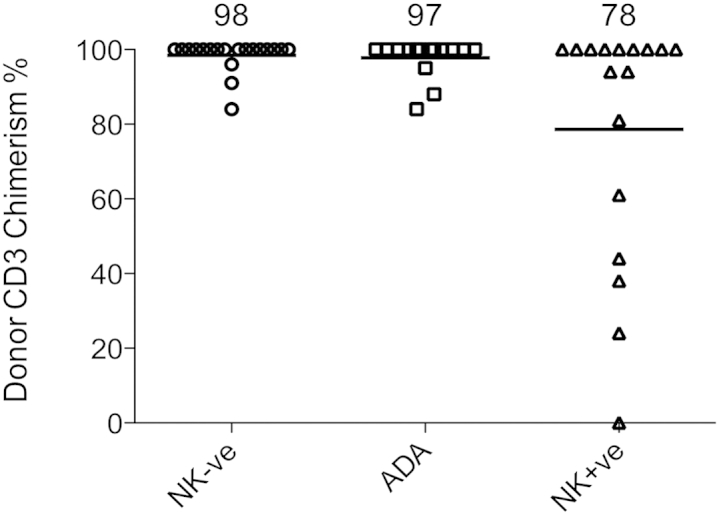
High levels of donor T-cell (CD3) engraftment were achieved in patients with NK^−^SCID and ADA-SCID, but a number of children with NK^+^SCID had mixed T-cell chimerism. *NK+ve*, NK^+^; *NK-ve*, NK^−^.

Using lineage-specific chimerism analysis, we found high levels of donor T-cell (CD3) engraftment in patients with NK^−^SCID (mean, 98% donor CD3) and ADA-SCID (mean, 97% donor CD3), but a number of children with NK^+^SCID (7/13 evaluated) had mixed T-cell chimerism (mean, 78% donor CD3; Fig 5 and see Table E1). One of these subjects with a donor T-cell chimerism of 61% was unusual in having had pretransplantation T-cell counts of greater than 1000 × 10^6^/L, although with an abnormal T-cell receptor repertoire and impaired PHA response. As expected, in the absence of preconditioning, B-cell (CD19, see Fig E3 in this article's Online Repository at www.jacionline.org) and myeloid (CD15) chimerism (see Fig E4 in this article's Online Repository at www.jacionline.org) in peripheral blood was limited to a small number of children across the groups. Replacement immunoglobulin therapy was withdrawn in 21 of 24 children with ADA-SCID, reflecting intact host B-cell development and function in the majority of these children once detoxification was established. In the NK^+^SCID group, 11 of 17 infants have continued to receive immunoglobulin replacement therapy. In the T^−^B^+^NK^−^SCID cohort immunoglobulin replacement therapy was successfully withdrawn in 12 of 22 children, including some children with no measurable donor B-cell chimerism, and here, host-derived B-cell immunity appears to have recovered once donor-derived reconstitution of T-cell help was established.

## Discussion

The first bone marrow transplantation for SCID was successfully performed without preconditioning by using whole, unmanipulated sibling donor marrow in 1968.^1^ Over the following 4 decades, there have been notable improvements in overall survival and long-term outcomes for the majority of infants undergoing allo-SCT for SCID, but there remains controversy over when grafts should be infused without conditioning and which procedures are best performed with conditioning. Overall, we found that survival after nonconditioned procedures was superior to that after conditioned grafts performed over the same period of time, but in the absence of directly matched cohort comparisons, little emphasis can be placed on this finding. Rather, our detailed analysis of outcome data from the nonconditioned group might help determine which SCID disorders are most amenable to correction in this manner and which subgroups would most likely benefit from preparative chemotherapy. Even then, important caveats must be considered while drawing conclusions, including the limited information available defining pretransplantation comorbidities in the NK^−^ and NK^+^ groups. In addition, we note the molecular heterogeneity in the NK^+^ group, with infants with known defects of VDJ recombination having poorer survival compared with the rest of the cohort, emphasizing the importance of genetic characterization for detailed outcome comparisons.

The absence of host T-cell immunity in patients with SCID disorders provide a unique environment, which is considered permissive for donor cell engraftment. Here we have considered the importance of host NK immunity as a barrier to donor engraftment and as an indicator of vacant thymic and prethymic niches receptive to donor cell engraftment. NK cells are capable of graft rejection, particularly in the nongenoidentical setting.^11^ In the context of patients with SCID, host NK cells might not only act as a barrier to transplantation by mediating graft rejection, but also their presence might indicate that stem cell or lymphocyte progenitor cell niches are occupied and nonreceptive to donor engraftment.

Most SCID disorders arise because of defective receptor rearrangement pathways, loss of cytokine receptor expression, or abnormal signaling pathways, which result in a block in T-cell and NK cell development. These conditions are defined by absent or functionally abnormal T cells, with variable loss of B cells and NK cell immunity. In rare cases there might be circulating T cells, which have “leaked,” but in general, these are usually poorly functional with a limited T-cell receptor repertoire and exhibit impaired mitogen responses. In most conditions, in the absence of conditioning, thymic and prethymic bone marrow stem cell niches remain occupied with host progenitors, and donor engraftment is restricted to the peripheral T-cell compartment. The absence of NK development in patients with SCID-X1 and JAK3 deficiency reflects a block in common lymphoid progenitor development affecting T and NK cells but not B cells. In these conditions the niche for T/NK common precursors is presumed vacant, and we speculate that prethymic niches, early thymic niches, or both are therefore receptive for donor T-cell precursor engraftment, allowing sustained donor derived thymopoiesis. In contrast, T-cell arrest in the majority of NK^+^SCID disorders probably arises during thymic ontogeny at later stages of committed T-cell differentiation when T-cell receptor or CD4/8 coreceptor signaling is required. Here, replete precursor niches are not receptive to engraftment, and unless preconditioning with chemotherapy is undertaken, there might be competition with donor precursors. Therefore long-term immune reconstitution in this context is largely dependent on postthymic T-cell recovery and is therefore poorer than in patients with NK^−^SCID disorders. ADA-SCID can be considered separately as a systemic metabolic disorder in which accumulation of toxic metabolites compromises lymphocyte and common lymphoid precursor. In this disorder donor T cells and prethymic stem cell progenitors engraft and mediate detoxification, leading to thymopoiesis and multilineage immune reconstitution.

The risks of immediate conditioning-related toxicity have to be balanced against later consequences that might arise as a result of slow or poor immune recovery after nonconditioned procedures. Chemotherapy preconditioning has evolved over time, and improvements include the availability of targeted dosing or the use of less toxic, reduced-intensity conditioning regimens.^15-17^ Nonetheless, in young infants there is a notable morbidity and mortality associated with conditioned procedures, and overall transplant-related mortality after such procedures is approximately 20% at our centers. In infants with established comorbidities, there might be heightened concerns relating to the tolerability of preparative regimens, and thus the alternative approach without conditioning might be much more attractive. Finally, in some families late effects, including infertility associated with myeloablative regimens, are a major concern, and the option of a nonconditioned infusion is often favored.

Earlier cohort analyses considered reconstitution and survival outcomes for SCID disorders based on T^−^B^−^ and T^−^B^+^ phenotypes, the latter faring better after nonconditioned infusions of either whole marrow in the genoidentical setting or hematopoietic stem cells in the T cell–depleted mismatched setting.^14^ Establishment of donor B-cell chimerism and recovery of B-cell function sufficient to withdraw immunoglobulin therapy has been reported to be variable and unpredictable in these children.^18^ In our cohorts there was no significant difference in survival between B^+^ and B^−^ SCID disorders.

**Fig 6:**
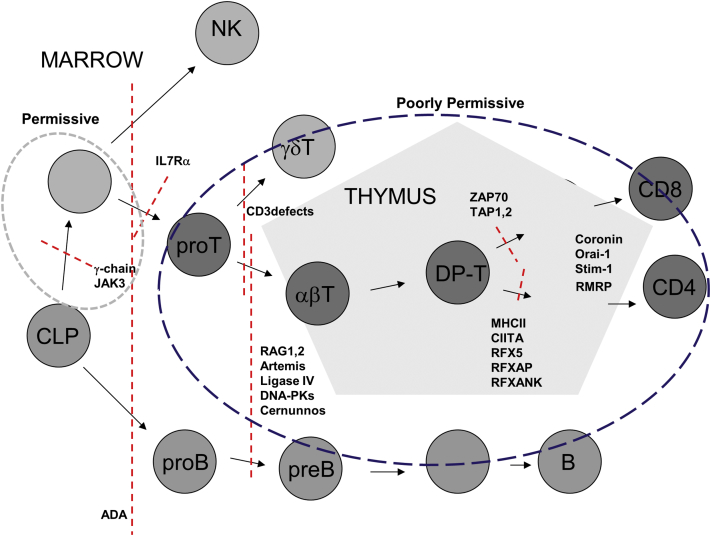
Phenotypic classifications of SCID are based on the presence or absence of T, B, and NK cells. The genetic basis of SCID disorders can now be elucidated in the majority of infants, although historically, transplantation has often proceeded before mutations could be identified. T^−^B^+^NK^−^ disorders arise after blocks in T and NK cell development, and we speculate that in these infants vacant and receptive bone marrow niches are receptive and permissive for engraftment of donor-derived precursors without conditioning. The presence of circulating NK cells is strongly indicative that common T/NK niches are occupied, and this might result in engraftment competition with donor precursors. Certain disorders with a T^−^B^+^NK^+^ phenotype (eg, IL-7 receptor deficiency and CD3 signaling defects) might be more permissive than T^−^B^−^NK^+^ disorders, perhaps reflecting an intermediate stage of T-cell (but not NK cell) developmental arrest. Here transplantation without conditioning might well be successful, but in other NK^+^ conditions, including defects of VDJ recombination, conditioning is likely to be required to clear niches and secure precursor engraftment. In the case of ADA-SCID, accumulation of toxic metabolites compromises all lymphoid lineages as shown, but detoxification after nonconditioned transplantation can be sufficient to ensure engraftment of long-term multilineage progenitors. *CIITA*, Class II transactivator; *CLP*, common lymphoid progenitor; *DNA-PK*, DNA protein kinases; *DP-T*, double positive T cells; *IL7Rα*, IL-7 receptor α; *RAG*, recombination-activating gene; *RFX5*, regulatory factor X5; *RFXANK*, regulatory factor X ankyrin repeat containing; *RFXAP*, regulatory factor X5 associated protein *RMRP*, mitochondrial RNA-processing endoribonuclease; *Stim1*, stromal interaction molecule 1; *TAP*, transporter associated with antigen processing; *ZAP70*, ζ chain–associated protein of 70 kDa.

The additional characterization of SCID phenotypes on the basis of NK activity provides a biological explanation for engraftment permissiveness, especially in the HLA-mismatched setting. Our findings broadly confirm improved survival and better T-cell recovery in patients with T^−^B^+^ SCID because many of these disorders are caused by defects of the common cytokine receptor γc or JAK3, which result in a T^−^B^+^NK^−^ phenotype. The molecular basis of NK^+^SCID disorders (Fig 6) is heterogeneous, and although not yet fully defined for a number of children reported here, included defects of VDJ recombination and radiation sensitivity disorders in which survival was poorer. The latter are usually T^−^B^−^NK^+^ and might not always be suitable for conventional conditioning regimens but can tolerate reduced-intensity preparations, which are capable of securing engraftment of bone marrow stem cells with multilineage potential. NK immunity is also preserved in IL-7 receptor defects (T^−^B^+^NK^+^); a number of these infants are reported to have successfully undergone unconditioned procedures, and some have recovered antibody production.

Overall, our findings suggest that NK^−^SCID disorders (including ADA-SCID) are highly permissive and receptive to allo-SCT, and conditioning is not required to secure satisfactory long-term T-cell immunity, particularly in the matched sibling/family donor setting. Longer-term follow-up is required to fully determine the quality and longevity of immune reconstitution, especially where ongoing immunoglobulin replacement is required. In patients with NK^+^SCID, a high proportion of infants required second procedures, and long-term T-cell recovery is generally less good. There is a strong argument that the host environment is less permissive in the majority of patients with NK^+^SCID disorders, and therefore preconditioning should be offered as the most effective way of securing engraftment as part of a first and single procedure. Of course, there might be situations in sick infants in which conditioning might not be tolerated, and a pragmatic approach could require immediate graft infusion without chemotherapy in the expectation that a second conditioned procedure might be required. Finally, identification of the molecular basis of these disorders before allo-SCT should allow further stratification within the groups and will help determine the need and intensity of preparative regimens (Fig 6).

**Clinical implications**

The detection of circulating NK cells in infants with SCID provides an indication that allogeneic transplantation should be performed with chemotherapy preconditioning.

## Supplementary data

Table E1

Online Figure Legends E1-E4
