# Correction

**DOI:** 10.1016/j.jaci.2016.02.003

**Published:** 2016-04

With regard to the article in the June 2014 issue entitled “Host natural killer immunity is a key indicator of permissiveness for donor cell engraftment in patients with severe combined immunodeficiency” (J Allergy Clin Immunol 2014;133:1660-6), the authors report an incorrect funding statement, which should read as follows: W.Q. and H.G. are supported by GOSHCC trustees. This research was supported by the National Institute for Health Research Biomedical Research Centre at Great Ormond Street Hospital for Children NHS Foundation Trust and University College London. The authors regret the error.

