# Supplementary material for: Host natural killer immunity is a key indicator of permissiveness for donor cell engraftment in patients with severe combined immunodeficiency
Source: J Allergy Clin Immunol. 2014 Jun;133(6):1660–6. doi: 10.1016/j.jaci.2014.02.042 (PMC4048544; doi:10.1016/j.jaci.2014.02.042)
Supplement: Online Figure Legends E1-E4 [file mmc2.docx]

**Online Repository: E-Figures**

**Host natural killer immunity is key indicator of permissiveness for donor cell engraftment in SCID**

**E Figure 1.** Previous studies have defined two groups of SCID disorder based on the presence or absence of B cells in peripheral blood at the time of diagnosis, However, we found no significant difference in survival for T^-^B^+^ and T^-^B^-^ SCID disorders following non-conditioned transplantation.

**E Figure 2**. There was no significant difference in CD4 immune recovery in infants transplanted below or above the age of 3 months.

**E Figure 3**. In the absence of pre-conditioning B cell (CD19) chimerism was limited

**E Figure 4.** Myeloid (CD15) chimerism was also limited to a minority of subjects, mostly in the ADA group.
