# Supplementary material for: Host natural killer immunity is a key indicator of permissiveness for donor cell engraftment in patients with severe combined immunodeficiency
Source: J Allergy Clin Immunol. 2014 Jun;133(6):1660–6. doi: 10.1016/j.jaci.2014.02.042 (PMC4048544; doi:10.1016/j.jaci.2014.02.042)
Supplement: Supplementary file 2 [file mmc1.docx]

E-Table 1. Patient Characteristics, transplant and outcome

Phenotype	Genetic diagnosis	Age Infused	Donor	Graft Type	GVHD Prophylaxis	GVHD	% Donor CD3	Ig withdrawal	Vaccinated	Outcome
	γ-chain	11.0	Haplo	PBSC	CyA	N/A	N/A	N/A	N/A	Dead
	γ-chain	0.0	MSD	BM	None	None	55*	Yes	Yes	Alive
	γ-chain	8.0	MSD	BM	None	Skin Stage II	100	Yes	Yes	Alive
	γ-chain	2.3	Haplo	PBSC	CyA	Skin Stage II	100	No	N/A	Alive
	γ-chain	8.0	MSD	BM	None	Skin Stage III	100	Yes	Yes	Alive
	γ-chain	8.0	MSD	BM	None	None	100	Yes	Yes	Alive
	γ-chain	0.8	MSD	BM	None	Skin Stage I	100	Yes	Yes	Alive
	γ-chain	3.7	MSD	BM	None	Grade III skin & gut	100	Yes	Yes	Alive
	γ-chain	1.4	Haplo	PBSC	CyA	None	100	Yes	Yes	Alive
	γ-chain	7.0	Haplo	PBSC	None	None	84	N/A	N/A	Dead
	γ-chain	1.5	MUD	Cord	None	Grade III skin & gut	100	Yes	Yes	Alive
	γ-chain	0.5	Haplo	PBSC	None	Skin Stage II	N/A	N/A	N/A	Dead
NK-ve SCID	γ-chain	2.0	Haplo	PBSC	None	None	100	Yes	Yes	Alive
	γ-chain	9.0	MUD	Cord	CyA & Pred	Grade II skin & gut	100	No	N/A	Alive
	γ-chain	5.0	MUD	BM	CyA	Grade II gut	100	Yes	Yes	Alive
	γ-chain	6.0	Haplo	BM	CYA	None	100	No	N/A	Alive
	**γ-chain**	**11.0**	**Haplo**	**BM**	**CYA**	**None**	**100**	**No**	**N/A**	**Alive**
	γ-chain	7.0	MSD	BM	None	Skin Stage II	100	No	N/A	Alive
	γ-chain	1.0	Haplo	PBSC	CyA	Grade II skin & gut	100	No	N/A	Alive
	γ-chain	8.0	MUD	Cord	CyA, MMF & Pred	chronic skin GVHD	100	No	N/A	Alive
	γ-chain	8.0	Haplo	BM	CyA & MMF	Grade III skin & Liver	100	No	N/A	Alive
	γ-chain	0.5	MSD	BM	CyA	Skin Stage II	96	No	N/A	Alive
	JAK 3	8.0	Haplo	PBSC	CyA	None	10*	No	N/A	Alive
	**JAK 3**	**10.0**	**Haplo**	**PBSC**	**CyA**	**Skin Stage II**	**100**	**No**	**N/A**	**Alive**
	Jak3	7.0	Haplo	BM	CyA	None	100	Yes	Yes	Alive
	Jak3	1.0	Haplo	BM	CyA & MMF	None	100	No	N/A	Alive
	Jak3	8.0	MSD	BM	None	None	91	Yes	Yes	Alive
	ADA	1.0	MSD	BM	None	None	85*	Yes	Yes	Alive
	ADA	3.8	MSD	BM	None	None	N/A	N/A	N/A	Dead
	ADA	1.8	MSD	BM	None	None	100	Yes	Yes	Alive
	ADA	6.0	MSD	BM	None	None	60*	Yes	Yes	Alive
	ADA	7.5	MSD	BM	None	None	100	Yes	Yes	Alive
	ADA	5.5	MFD	BM	None	None	62*	Yes	Yes	Alive
	ADA	3.0	MFD	BM	CSA	None	100	Yes	Yes	Alive
	ADA	3.0	MSD	BM	None	None	95	Yes	Yes	Alive
	ADA	1.0	MSD	BM	None	None	N/A	N/A	N/A	Dead
	ADA	2.5	MSD	BM	CyA	Grade III gut	100	Yes	Yes	Alive
	ADA	4.0	MFD	BM	CyA MMF	Grade III skin & gut	100	Yes	N/A	Alive
	ADA	2.5	MSD	BM	None	Grade II skin & gut	100	yes	Yes	Alive
	ADA	1.5	MSD	BM	None	None	100	No	N/A	Alive
	**ADA**	**16.0**	**MSD**	**BM**	**None**	**None**	**100**	**Yes**	**N/A**	**Alive**
	ADA	16.0	MSD	Cord	CYA	None	100	No	N/A	Alive
	**ADA**	**30.0**	**MSD**	**BM**	**CYA**	**None**	**100**	**Yes**	**Yes**	**Alive**
	ADA	3.0	MSD	BM	CyA	None	100	Yes	Yes	Alive
	ADA	5.0	MFD	BM	CyA	None	100	Yes	Yes	Alive
	ADA	2.5	MFD	BM	CyA	None	100	Yes	Yes	Alive
	ADA	17.0	MSD	BM	None	None	84	Yes	yes	Alive
	ADA	1.0	MSD	BM	None	Skin stage II	100	Yes	Yes	Alive

E-Table 1. Patient Characteristics, transplant and outcome
